# Beyond filtration: A kidney support therapy framework for interpreting renal replacement therapy timing evidence

**DOI:** 10.1016/j.jointm.2026.01.003

**Published:** 2026-03-01

**Authors:** Sanat Subhash, Rupesh Raina

**Affiliations:** 1 Northeast Ohio Medical University, Rootstown, USA; 2 Akron Nephrology Associates/Cleveland Clinic Akron General Medical Center, Akron, USA

The perspective paper *Timing of initiating renal replacement therapy in acute kidney injury* by Barbar et al.^[^^1^^]^ offers a thorough synthesis of evidence guiding the timing of renal replacement therapy (RRT) in acute kidney injury, providing a useful starting point for broader discussion.^[^^1^^]^ However, the analysis is rooted in a framework that views RRT primarily as a filtration replacement modality. This narrow interpretation does not reflect the broader clinical reality in which extracorporeal therapies are used across the intensive care unit to stabilize physiology, manage systemic deterioration, and support failing organs. We propose a shift from “kidney replacement therapy” to “kidney support therapy” (KST), a terminology change that more accurately reflects the clinical reality in which extracorporeal therapy stabilizes physiology, supports failing organs, and mitigates systemic injury. The conceptual framework underlying this KST approach was developed by the authors and is presented as a proposed clinical decision framework (Supplementary Figure S1). Reconsidering timing through the lens of KST reveals why many critically ill patients fall outside the traditional early versus late approach-delayed RRT paradigm.

Barbar et al.^[^^1^^]^ report that early initiation of RRT does not improve mortality in severe acute kidney injury when urgent complications are absent, with many patients in delayed-treatment arms recovering kidney function without requiring dialysis. These findings are statistically validated within classic AKI populations.

However, the populations excluded from these timing trials, many of which face significant comorbidities and mortality risk, are those in which modern clinicians initiate extracorporeal therapy for nonrenal reasons.

Raina et al.^[^^2^^]^ describe a variety of kidney therapies frequently initiated in septic shock, hematopoietic stem cell transplantation (HSCT)-related complications, veno-occlusive disease, cardiopulmonary bypass, tumor lysis syndrome, chemotherapy toxicity, and hyperammonemia, situations where organ dysfunction develops before overt renal failure. In these contexts, timing decisions are driven by systemic deterioration rather than strictly by creatinine or urine output. The relevant timing threshold is the onset of systemic instability, not the attainment of Kidney Disease: Improving Global Outcomes-defined renal failure. The KST framework emphasizes initiating support early enough to blunt the escalating burden of cytokines, toxins, fluid accumulation, or hemodynamic stress before they precipitate multiorgan dysfunction. Extracorporeal therapy in these settings provides hemodynamic stabilization, solute control, cytokine modulation, toxin removal, and volume management, functioning as multiorgan support.^[^^2^^]^ These functions extend well beyond the replacement of filtration, supporting the argument for adopting the KST terminology.

The impact of broader KST implementation also occurs within pediatric patients, where critically ill or immunosuppressed children often experience renal injury alongside multiorgan dysfunction, with extracorporeal therapy used as supportive therapy across these systemic complications.^[^^2^^]^ As a result, timing is guided by the goal of averting physiological collapse instead of relying on late-stage biochemical evidence of AKI to trigger intervention.

Pediatric patient experience with sinusoidal obstruction syndrome (SOS) after hematopoietic cell transplantation also presents opportunities for KST implementation. SOS carries very high mortality and frequently presents with diuretic-resistant fluid overload and stage Ⅲ AKI, prompting early continuous renal replacement therapy as part of a standardized supportive care protocol.^[^^3^^]^ In a 34-patient cohort, early continuous renal replacement therapy (CRRT) initiation within roughly 8 to 12 hours of meeting criteria led to the resolution of SOS symptoms in four of six children.^[^^3^^]^ Extracorporeal therapy served as systemic support rather than replacement therapy in this population, demonstrating the broader implementation of this treatment methodology.

Fluid overload is a central determinant of timing in pediatrics, and fluid overload of 20% or more at CRRT initiation is associated with significantly increased mortality.^[^^4^^]^ Thus, early initiation can be life-preserving long before creatinine levels would mandate classic replacement therapy within critically ill populations. Additional pediatric and adult data reinforce this effect, with each day of delay before CRRT initiation increasing the likelihood of major adverse kidney events at ninety days in children and young adults.^[^^5^^]^ Taken together, these observations support the KST framework in which earlier initiation is sometimes necessary to prevent systemic collapse, not merely to replace renal clearance.

When interpreted through this lens, the conclusions regarding early *vs*. delayed initiation by Barber et al.^[^^1^^]^ apply to classic AKI but cannot be extended to the heterogeneous ICU populations in which extracorporeal support is deployed for systemic instability rather than renal failure alone.

Their trial populations exclude septic shock, HSCT complications, metabolic emergencies, and pediatric multiorgan failure, all of which depend on supportive extracorporeal therapy. The question of timing shifts from replacing filtration at a biochemically defined threshold to interrupting a trajectory of physiologic failure. This distinction underlies why support-based initiation follows a different logic than replacement-based initiation and why traditional timing frameworks cannot adequately guide practice in these high-risk groups.

As intensive care practice continues to evolve, KST provides a more accurate structure for decision-making. It aligns extracorporeal therapy with its modern goals: stabilizing hemodynamics, controlling solute burden, managing fluid overload, supporting metabolism, and preventing irreversible organ injury. Reframing therapy in this way clarifies why timing strategies differ across populations and why support-based initiation is critical in pediatrics and systemic injury syndromes. Adopting the KST terminology and timing methodology would strengthen communication, align clinical practice with physiologic intent, and better contextualize timing recommendations across the ICU.

## Acknowledgements

None.

## Funding

None.

## Ethics Statement

Not applicable.

## Conflicts of Interest

The authors declare that they have no known competing financial interests or personal relationships that could have appeared to influence the work reported in this paper.

## Data Availability

Not applicable.

## CRediT authorship contribution statement

**Sanat Subhash:** Formal analysis, Data curation, Conceptualization. **Rupesh Raina:** Investigation, Funding acquisition, Formal analysis, Data curation.

## Appendix Supplementary materials

Supplementary Figure: Conceptual decision pathway for assessment, initiation, and treatment deescalation of continuous renal replacement therapy (CRRT) in acute kidney injury. The figure integrates acute kidney injury phenotypes, biomarker-based risk stratification, standard and support-driven indications for CRRT initiation, and criteria for CRRT stepdown and transition to intermittent modalities, promoting a kidney support therapy framework within critical illness. Image, image 1 dummy alt text
