# Supplementary figures and images for: Beyond filtration: A kidney support therapy framework for interpreting renal replacement therapy timing evidence
Source: J Intensive Med. 2026 Mar 1;6(4):349–50. doi: 10.1016/j.jointm.2026.01.003 (PMC13323552; doi:10.1016/j.jointm.2026.01.003)

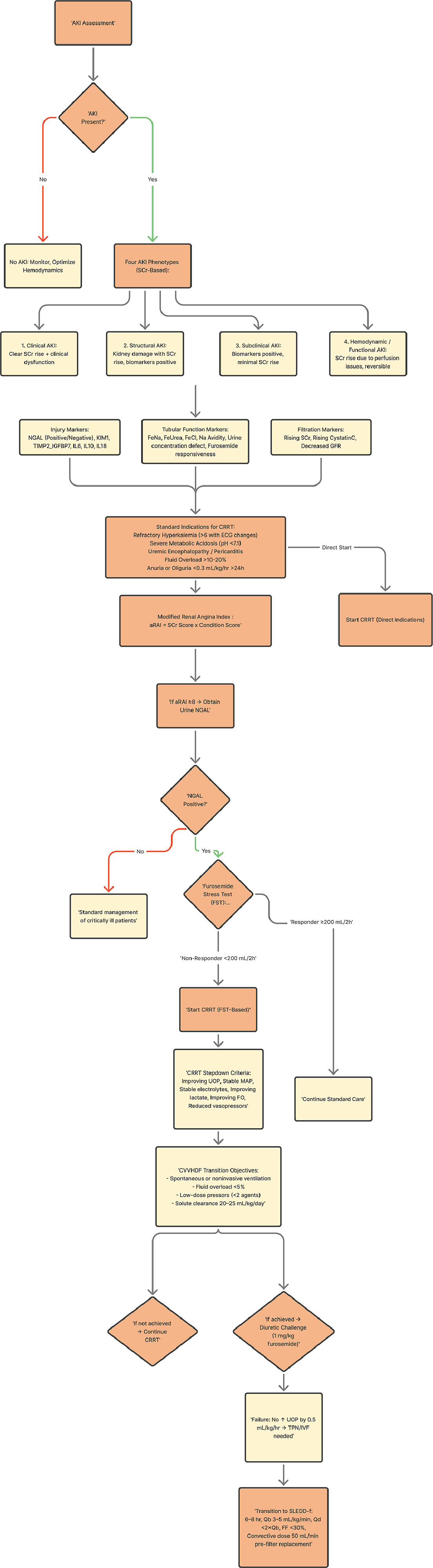

Supplement: Supplementary file 1 — Supplementary Figure: Conceptual decision pathway for assessment, initiation, and treatment deescalation of continuous renal replacement therapy (CRRT) in acute kidney injury. The figure integrates acute kidney injury phenotypes, biomarker-based risk stratification, standard and support-driven indications for CRRT initiation, and criteria for CRRT stepdown and transition to intermittent modalities, promoting a kidney support therapy framework within critical illness. [file mmc1.jpg]
